# Immune-enhancing effects of anionic macromolecules extracted from *Codium fragile* on cyclophosphamide-treated mice

**DOI:** 10.1371/journal.pone.0211570

**Published:** 2019-02-19

**Authors:** Chaiwat Monmai, SangGuan You, Woo Jung Park

**Affiliations:** Department of Marine Food Science and Technology, Gangneung-Wonju National University, Gangneung, Gangwon, Korea; Center for Cancer Research, UNITED STATES

## Abstract

Immune-regulation and homeostasis are critical in cancer therapy and immunomodulatory biomaterials have been used to decrease side effects of immunosuppressant drugs. Anionic macromolecules (CFAMs) were isolated from the seaweed *Codium fragile*, and its immune-enhancing biological activities were examined in CY-induced immunosuppressed mice. CFAMs improved the splenic lymphocyte proliferation, NK cell activity, and spleen index. The expression of immune-associated genes was highly upregulated in splenic lymphocytes, and gene expression was differently regulated according to mitogens such as T-cell (Con A) and B-cell (LPS) mitogens. Additionally, CFAMs boosted the proliferation, NO production, and phagocytosis of peritoneal macrophages. CFAMs also considerably stimulated immune-associated gene expression in peritoneal macrophages. Moreover, our results showed CFAMs mediated its immune-enhancing effects via the MAPK pathway. These suggested CFAMs can be used as a potent immunomodulatory material under immune-suppressive condition. Furthermore, CFAMs may also be used as a bio-functional and pharmaceutical material for improving human health and immunity.

## Introduction

Immunomodulation, consisting of immune-stimulation and immunosuppression, is a sophisticated mechanism regulating the pathophysiology and etiology of various diseases affecting the immune system [1]. Immuno-regulation and homeostasis are crucial factors affecting cellular processes [2]. An immunomodulatory material can be used as an immune-stimulator to decrease the side effects of immunosuppressant drugs [3]. From ancient times, natural products with immune-regulatory properties have been considered to be important. They also act as important nutrients for improving human health and preventing chronic diseases and physiological problems, such as allergies, infections, and autoimmune diseases. Beta-glucan, a polysaccharide, is useful for treating respiratory tract infections [4]. Hemicellulose is known to possess immune-regulatory, antibacterial, and antitumor biological activities [5]. Chitin also enhances innate immunity and modulates inflammation in occupational allergies in humans [6]. Moreover, carbohydrates are known to be associated with the regulation of gut microbiota, which affect host immunity and health [7].

Most anticancer chemotherapeutic drugs, such as cyclophosphamide (CY), gemcitabine, and 5-fluorouracil, kill cancer cells; however, they also destroy normal cells, including immune cells, during the cytokine storm [8]. CY is a cancer prodrug and its therapeutic effectiveness depends on its metabolism by cytochrome P450 (CYP). It is the most widely used alkylating agent in chemotherapy for treating several cancers [9]. It acts on immune cells (such as T and B cells) which are xenoreactive, and thus, proliferate only after antigen stimulation [10]. The antitumor effect of CY is proportional to the amount of CY administered, and often leads to immunosuppression and cytotoxic effects [11]. Biomaterials that reduce the side effects of anticancer drugs are important for improving the quality of life of cancer patients [12].

*Codium fragile* is a popular, edible green alga belonging to the family *Codiaceae* It is widely distributed along the shores of East Asia, Oceania, and Northern Europe. *C*. *fragile* has also been used as an oriental medicine for treatment of diseases, such as enterobiosis, dropsy, and dysuria [13]. *C*. *fragile* extracts have been demonstrated to possess thrombolytic, anticoagulant, antiplatelet [14], anti-inflammatory [15], and immune-stimulatory activities on murine macrophage cells via the activation of NF-κB and MAPK pathways [16].

Recently, the crude anionic macromolecules from *C*. *fragile* (CFAMs) were extracted and their immunomodulatory activities was demonstrated on macrophages and NK cells in addition to chemical compositional analysis [16–18]. However, no previous study reports their immune-enhancing regulation activity using in vivo immune-suppressed animal physiological system. We hypothesize that CFAMs may have immune-enhancing activity in immune organs and cells such as spleen, NK cells, and peritoneal macrophages under *in vivo* immunosuppressed animal physiological condition. Herein, the present study induced immunosuppressed condition of in vivo mice using CY and investigated the immune-enhancing effects through oral administration of CFAMs on CY-induced immunosuppressed mice as well as analyzed the immune signaling pathways involved in mediating those effects.

## Materials and methods

### Animals

Six-week-old inbred male BALB/c mice weighing 23 g were obtained from Central Lab. Animal Inc. (South Korea). These animals were kept in pathogen-free, environmentally controlled rooms maintained at 22 ± 2°C temperature and a 12-h dark–light cycle, for at least a week before the start of the experiment. They were fed on standard laboratory diet and water. All experimental procedures were approved by the Gangneung-Wonju National University committee for animal experiments (Approval number: GWNU-2016-31).

### Isolation of the polysaccharides

Extraction and purification of crude anionic macromolecules from *C*. *fragile* (CFAMs) were performed as did previously [16] and these CFAMs were used in this study. Briefly, CFAMs were extracted from the milled sample of *C*. *fragile* using EtOH and distilled water, following centrifugation, filtration, and evaporation after removal of proteins [19].

### Induction of immunosuppression in mice

Mice were randomly divided into seven groups (n = 5), after acclimatizing them for one week. One group was designated as the control group (normal group) and was administrated saline orally. The other groups were orally administrated saline (saline group) supplemented with varying concentrations of CFAMs (50, 100, 250, and 500 mg/kg BW) or with 100 mg/kg BW of commercial ginseng syrup (ginseng group). All groups received the respective treatment once per day for 10 consecutive days. At day 4–6 post-administration, mice (except those in the normal group) were injected intraperitoneally once a day with CY (80 mg/kg BW; Sigma–Aldrich, USA), and all the mice were sacrificed 24 h after completion of the treatment regimen.

### Preparation of peritoneal macrophages and splenocytes

Peritoneal macrophages were prepared using the Ray and Dittel method [20]. Five milliliters of ice-cold phosphate buffered saline (PBS, supplemented with 3% FCS) was injected into the peritoneal cavity of each mouse, and subsequently the macrophages were collected. After collection, the cell suspension was centrifuged and cell pellet were resuspended in the RPMI-1640 medium or PBS for cell counting.

Splenocytes were isolated from the spleen of BALB/c mice. The spleen was weighed and collected in ice-cold PBS. After treating the spleen with 1 × RBC Lysis Buffer (eBioscience, USA), the lysate cells were centrifuged at 400 × *g* for 10 min and washed using PBS. The splenocytes were resuspended in RPMI-1640 growth medium supplemented with 10% fetal bovine serum, streptomycin (100 μg/mL), and penicillin (100 IU/mL). Spleen index was evaluated using the following formula:

$$\mathrm{Spleen}\;\mathrm{index}=\frac{\mathrm{spleen}\;\mathrm{weight}\;(\mathrm{mg})}{\mathrm{mouse}\;\mathrm{body}\;\mathrm{weight}\;(g)}$$

### Determination of peritoneal macrophage proliferation and nitric oxide production

In order to determine the proliferation of macrophages, the EZ-Cytox Cell Viability Assay kit (Daeil Labservice, Korea) was used, as described previously [21]. Macrophage proliferation ratio (%) was analyzed using the following formula:

$$\mathrm{Macrophage}\;\mathrm{proliferation}\;\mathrm{ratio}\;(\%)=\frac{\mathrm{Absorbance}\;\mathrm{of}\;\mathrm{the}\;\mathrm{test}\;\mathrm{group}}{\mathrm{Absorbance}\;\mathrm{of}\;\mathrm{the}\;\mathrm{control}\;\mathrm{group}}x\;100$$

The immune-enhancing activity was measured based on nitric oxide (NO) production by macrophages. The cells were cultured with or without LPS, and NO concentration was measured using the Griess reagent (Sigma–Aldrich, USA) [22].

### Phagocytosis of peritoneal macrophages

Phagocytosis of peritoneal macrophages was determined using the neutral red uptake method, as described previously [23]. Macrophages were seeded in triplicates in microplates and grown overnight in complete RPMI-1640 medium at 37°C in a humidified atmosphere of 5% CO_2_. Phagocytosis of macrophages was measured using a microplate reader [absorbance (A), 540 nm]. Phagocytosis ratio was calculated using the following formula

$$\mathrm{Phagocytosis}\;\mathrm{ratio}\;\left(\%\right)=A_{\mathrm{test}}/A_{\mathrm{normal}}\times 100$$

### Proliferation of splenic lymphocytes

Isolated splenic lymphocytes were induced using a T-cell (Con A, 5 μg/mL) or B-cell (LPS, 10 μg/mL) mitogen. Cells in the negative control group were incubated in RPMI at 37°C in a humidified atmosphere of 5% CO_2_. After culturing for 48 h, cell proliferation was evaluated using the EZ-Cytox Cell Viability Assay kit.

### Cytotoxicity assays for measuring the natural killer cell activity of splenocytes

Natural killer cell activity of splenocytes (effector cells) was analyzed using YAC-1 cells (target cells) [24] and lactate dehydrogenase (LDH) solution (Promega Co., USA) [25, 26]. Splenocyte suspensions were added to the YAC-1 cells to obtain an effector-to-target cell ratio of 50:1. After 4 h incubation, the culture supernatants were mixed with a LDH solution. Absorbance was measured at 490 nm and the percentage of NK cell cytotoxicity was evaluated using the following formula: Cytotoxicity (%) = [(Experimental release-Spontaneous release)/(Maximum release-Spontaneous release)] × 100.

### RNA isolation and reverse transcription

Total RNA was prepared from the LPS-stimulated peritoneal macrophages and mitogen-stimulated (Con A and LPS) splenic lymphocytes using the Tri reagent^®^ (Molecular Research Center, Inc., USA). After isolation, RNA was dissolved in nuclease-free water and its concentration was analyzed using a nanophotometer (Implen, Germany). cDNA was synthesized from the extracted RNA using the High Capacity cDNA Reverse Transcription kit (Applied biosystems, USA), according to the manufacturer’s instructions.

### Analysis of immune gene expression using real-time PCR

Quantification of immune gene expression in peritoneal macrophages and splenocytes was performed using the QuantStudio™ 7 FlexReal-Time PCR System (ThermoFisher scientific, USA) and SYBR® Premix Ex Taq™ II (Takara Bio Inc., Japan). Relative gene expression was calculated using the 2^-ΔΔC^_T_ method [27] and β-Actin as the reference gene (Table 1).

**Table 1:** Oligonucleotide primers used in real-time PCR for evaluating immune gene expression.

Gene	Accession No.	Oligonucleotide Sequence
*IL-1β*	NM_008361.4	Fa: GGCCTCAAAGGAAAGAATC
		Rb: ACCAGTTGGGGAACTCTGC
*IL-2*	NM_008366.3	Fa: CCTGAGCAGGATGGAGAATTACA
		Rb: TCCAGAACATGCCGCAGAG
*IL-4*	NM_021283.2	Fa: ACAGGAGAAGGGACGCCAT
		Rb: GAAGCCCTACAGACGAGCTCA
*IL-6*	NM_031168.2	Fa: AGTTGCCTTCTTGGGACTGA
		Rb: CAGAATTGCCATTGCACAAC
*IL-10*	NM_010548.2	Fa: TACCTGGTAGAAGTGATGCC
		Rb: CATCATGTATGCTTCTATGC
*IFN-γ*	NM_008337.3	Fa: CTCAAGTGGCATAGATGT
		Rb: GAGATAATCTGGCTCTGCAGGATT
*TNF-α*	D84199.2	Fa: ATGAGCACAGAAAGCATGATC
		Rb: TACAGGCTTGTCACTCGAATT
*TLR-4*	NM_021297.3	Fa: CGCTCTGGCATCATCTTCAT
		Rb: GTTGCCGTTTCTTGTTCTTCC
*COX-2*	NM_011198.4	Fa: AGAAGGAAATGGCTGCAGAA
		Rb: GCTCGGCTTCCAGTATTGAG
*iNOS*	BC062378.1	Fa: TTCCAGAATCCCTGGACAAG
		Rb: TGGTCAAACTCTTGGGGTTC
*β-Actin*	NM_007393.5	Fa: CCACAGCTGAGAGGAAATC
		Rb: AAGGAAGGCTGGAAAAGAGC

F^a^ = Forward primer

R^b^ = Reverse primer

### Western blotting for analyzing the NF-κB and MAPK signaling pathways

RAW264.7 cells were cultured in different concentrations of CFP (1.875, 3.75, 7.5, or 15 μg/mL) or 1 μg/mL of LPS. Using RIPA buffer (Tech & Innovation, China), the cells were lysed and protein was extracted. Protein concentration was measured using the Pierce™ BCA Protein Assay kit (Thermo Scientific, USA). After resolving the proteins using SDS-polyacrylamide gel electrophoresis (SDS-PAGE), they were transferred on to a polyvinylidene fluoride (PVDF) membrane. Western blotting was performed as described previously [28]. using antibodies specific for p-NF-κB p65 (R&D systems), p-IκBα (Cell Signaling Technology), p-p38 (R&D systems), p-ERK1/2 (R&D systems), p-JNK (R&D systems), and α-tubulin (Abcam). Target proteins were detected using the Pierce® ECL Plus Western Blotting Substrate (Thermo Scientific, USA). Imaging was performed using the ChemiDoc XRS+ imaging system (Bio-Rad) and protein band intensities were analyzed using the ImageLab software (version 4.1, Bio-Rad).

### Effect of NF-κB and MAPK activation inhibitors on TNF-α expression

In order to evaluate the effects of NF-κB and MAPK activation inhibitors (such as ERK, JNK, and p38) on TNF-α expression, RAW264.7 cells were used. RAW264.7 cells were treated with 100 nM of NF-κB activation inhibitor (Merck, 481406), and 20 μM of ERK activation inhibitors (Merck, 328006), JNK (Merck, 420119), and p38 (Merck, 559389) [29]. After that, these cells were stimulated with varying concentrations of CFP (1.875, 3.75, 7.5, and 15 μg/mL) or 1 μg/mL of LPS. After extraction of total RNA, TNF-α expression was analyzed using the QuantStudio™ 7 FlexReal-Time PCR System, as described above.

### Statistical analysis

Statistical analysis was performed using the Statistix 8.1 software. Data were analyzed using one-way ANOVA followed by comparison with the saline or ginseng group. Differences between groups were considered to be statistically significant at *P < 0*.*05* and *P < 0*.*01*.

## Results

### Effect of CFAMs treatment on spleen index

**Fig 1:**
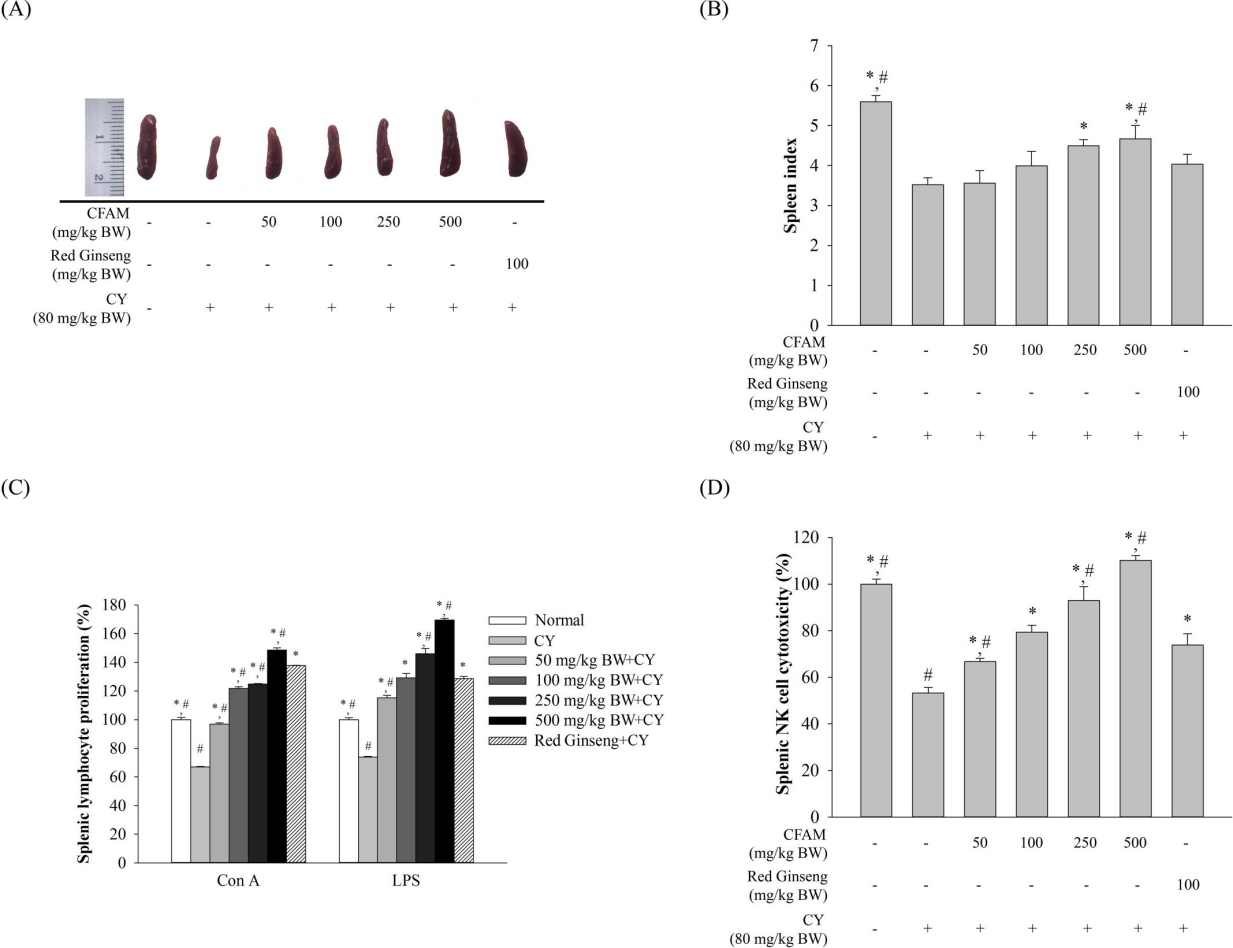
Effects of various concentrations of *Codium fragile* extracts. The spleen was collected from each group of mice (A). The spleen weight was divided with body weight for calculating the spleen index (B). The splenocytes were isolated and were stimulated with mitogens for splenic lymphocyte proliferation assay (C). The splenocytes were co-cultured with YAC-1 cells for splenic NK cells cytotoxic activity (D). Data were observed to be significantly different compared to the saline group (*, *P < 0*.*01*) and ginseng group *(#*, *P < 0*.*05*).

Fig 1A and 1B show the spleen size and spleen index of CY-induced mice treated with four different concentrations of CFAMs, respectively. Analysis was performed on the day of sacrifice. Compared to the normal group, the CY-treated group showed a marked reduction in the spleen index and spleen size. Compared to the CY group, spleen index of the group treated with 250 or 500 mg/kg BW CFAMs was significantly different; however, no significant differences in the body weight were observed between these groups (S1 Fig). Further, significant differences were not observed in the spleen size and spleen index of the group treated with 50 or 100 mg/kg BW CFAMs and the CY group.

### Effect of CFAMs treatment on splenic lymphocyte proliferation

Splenocytes from each group were incubated with two mitogens, namely Con A and LPS. Con A and LPS stimulate the T and B cells in splenocytes, respectively. Fig 1C shows that compared to the normal group, the proliferative response of splenic lymphocytes to both the T- and B-cell mitogens decreased markedly in the CY-treated group. However, compared to the CY-treated group, the CFAMs-treated group showed enhanced T and B cell proliferation in a dose-dependent manner. Further, the proliferation in the CFAMs-treated group was similar to that observed in the red ginseng-treated group, and significantly higher than that observed in the normal group.

### Effect of CFAMs on NK cell activity of splenocytes

In order to evaluate the NK cell activity of splenocytes, the splenocytes were co-cultured with YAC-1 cells. Cytotoxicity was determined using the LDH assay. Fig 1D shows that CY treatment suppressed splenic NK cell activity. However, CFAMs treatment gradually stimulated the NK cell activity of splenocytes. Notably, compared to the red ginseng-treated and normal groups, higher splenic NK cell activity was observed in the group treated with a high dose of CFAMs.

### Effect of CFAM on immune-associated gene expression in splenic lymphocytes

**Fig 2:**
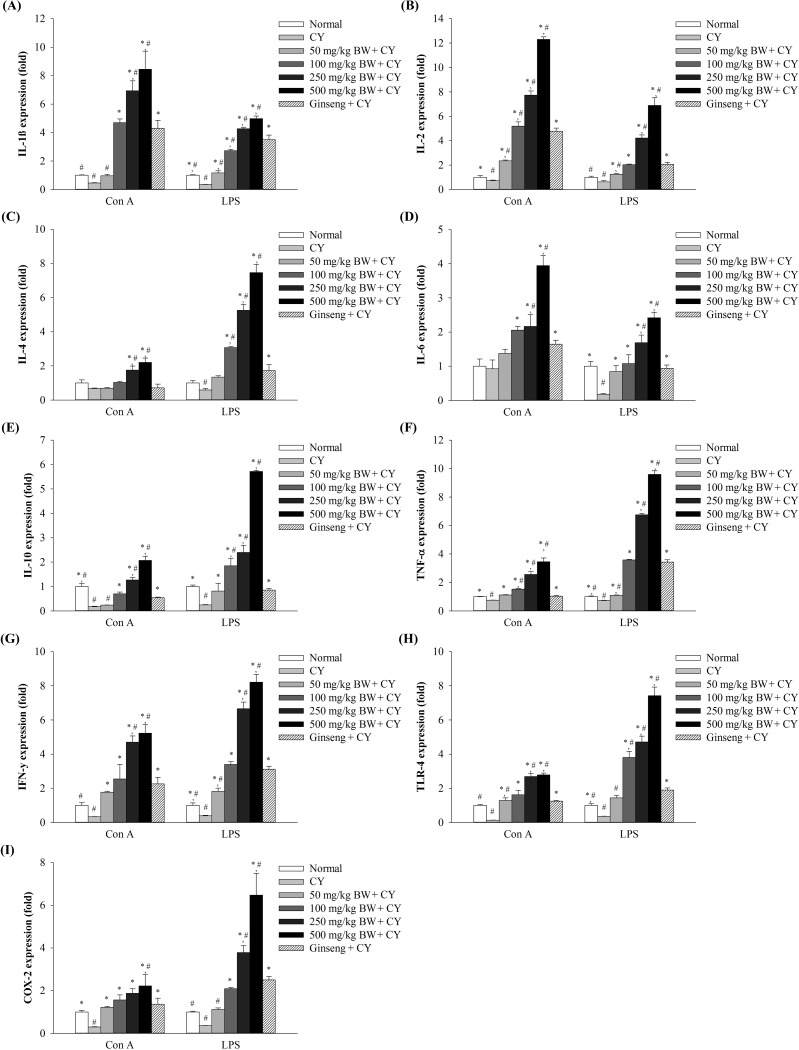
Relative expression (fold-change) of immune genes in mitogen-stimulated splenic lymphocytes. Splenic lymphocytes were stimulated with the mitogens (5 μg/mL of Con A or 10 μg/mL of LPS). After 48 h stimulation, total RNA was extracted from the stimulated cells. The cytokine expression was analyzed using the QuantStudio™ 7 FlexReal-Time PCR System. (A) IL-1β, (B) IL-2, (C) IL-4, (D) IL-6, (E) IL-10, (F) *TNF-α*, (G) *IFN-γ*, (H) *TLR-4*, and (I) *COX-2* genes. Significant differences were observed at *P < 0*.*05* compared with the saline (*) and ginseng (#) group.

By analyzing the expression of immune-associated genes (including those for cytokines) using qRT-PCR, the immuno-enhancing effects of CFAMs were elucidated. Fig 2 shows that compared to the normal group, the CY-treated group showed reduced expression of immune-associated genes in the spleen lymphocytes. However, treatment with varying concentrations of CFAMs enhanced immune-associated gene expression in splenic lymphocytes, which responded to the T- and B-cell mitogens. After CFAMs treatment, genes such as *IL-4*, *IL-10*, *TNF-α*, *IFN-γ*, *TLR-4*, and *COX-2* in splenic lymphocytes were highly upregulated and responded more to the B cell mitogen (LPS) than to the T cell mitogen (Con A). In contrast, the expression of *IL-1β*, *IL-2*, and *IL-6* was considerably higher in the Con A-stimulated cells than in the LPS-stimulated cells.

### Effect of CFAMs treatment on peritoneal macrophage proliferation and nitric oxide production

**Fig 3:**
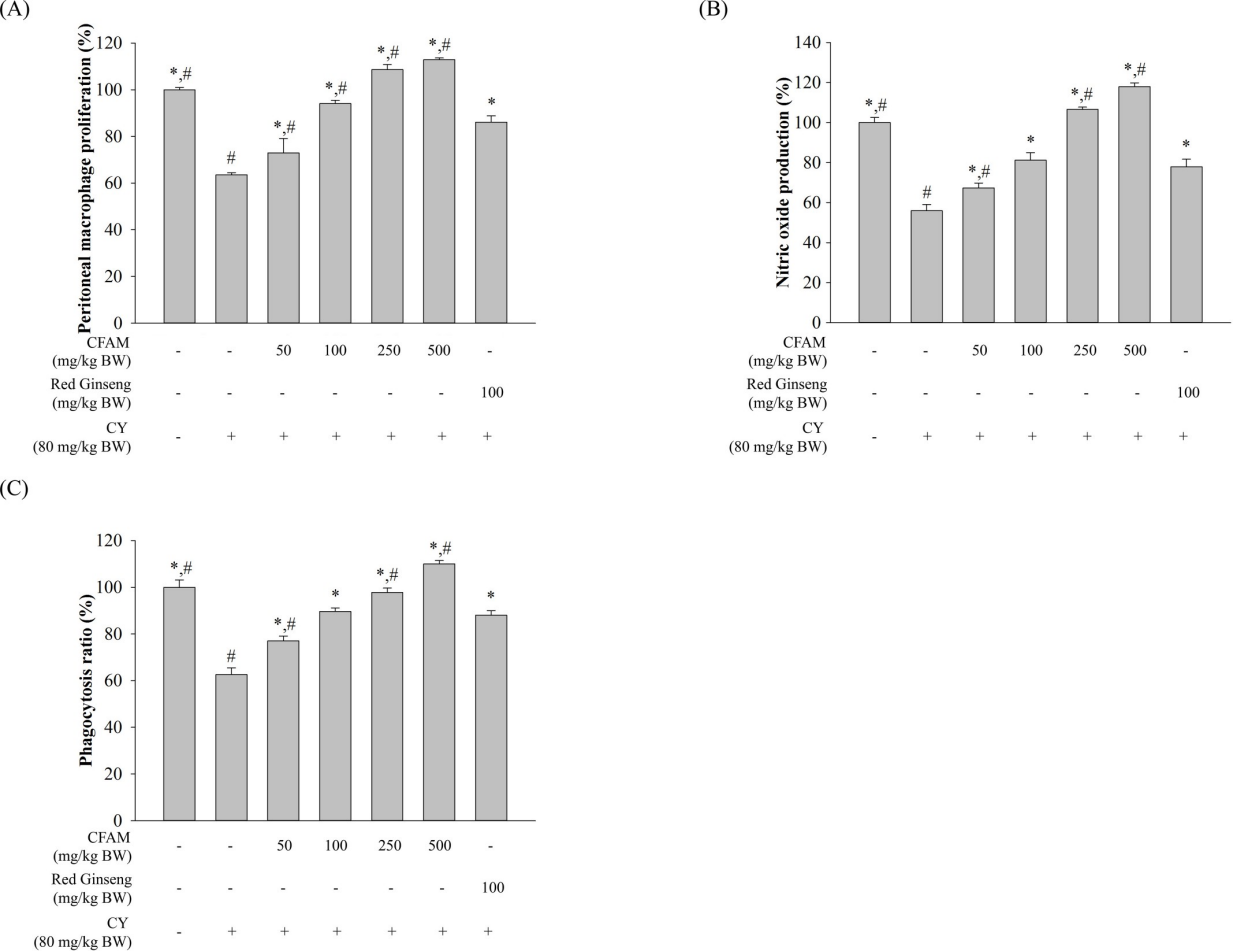
Effects of different concentrations of CFAMs on peritoneal macrophage. Peritoneal macrophages were isolated from each group of mice in the sacrifice day. The cells were stimulated with or without 1 μg/mL of LPS. LPS was used to investigate the immune response of macrophages isolated from each group. After 24h stimulated, the stimulated-cells were used for the peritoneal macrophage proliferation assay (A), and the cultured medium was used for the nitric oxide production assay (B). Phagocytosis of peritoneal macrophages was determined using the neutral red uptake (C). Significant differences were observed at *P < 0*.*01* compared with the saline group (*) and at *P < 0*.*05* compared with the ginseng group (#).

In order to investigate the effect of CFAMs on macrophage proliferation and nitric oxide production, peritoneal macrophages were isolated from each group of mice and stimulated using LPS. As shown in Fig 3A and 3B, compared to the CY-treated mice, the CFAMs-treated mice displayed a significant and dose-dependent increase in peritoneal macrophage proliferation and nitric oxide (NO) production. At high doses (250–500 mg/kg BW), CFAMs restored peritoneal macrophage proliferation and nitric oxide production. These restored levels were similar to those observed for the non-treated mice (normal group), and similar or higher than those observed for the ginseng-treated group.

### Effect of CFAMs on peritoneal macrophage phagocytosis

The neutral red uptake method has been used for the evaluation of phagocytosis by mouse peritoneal macrophages. Fig 3C shows that peritoneal macrophage phagocytosis was significantly lower in the CY group than in the normal group. Compared to the CY-treated group, peritoneal macrophage phagocytosis rates were remarkably and dose-dependently higher in the CFAMs-treated group. High doses of CFAMs also induced the recovery of peritoneal macrophage phagocytosis.

### Effect of CFAMs treatment on immune-associated gene expression in peritoneal macrophages

**Fig 4:**
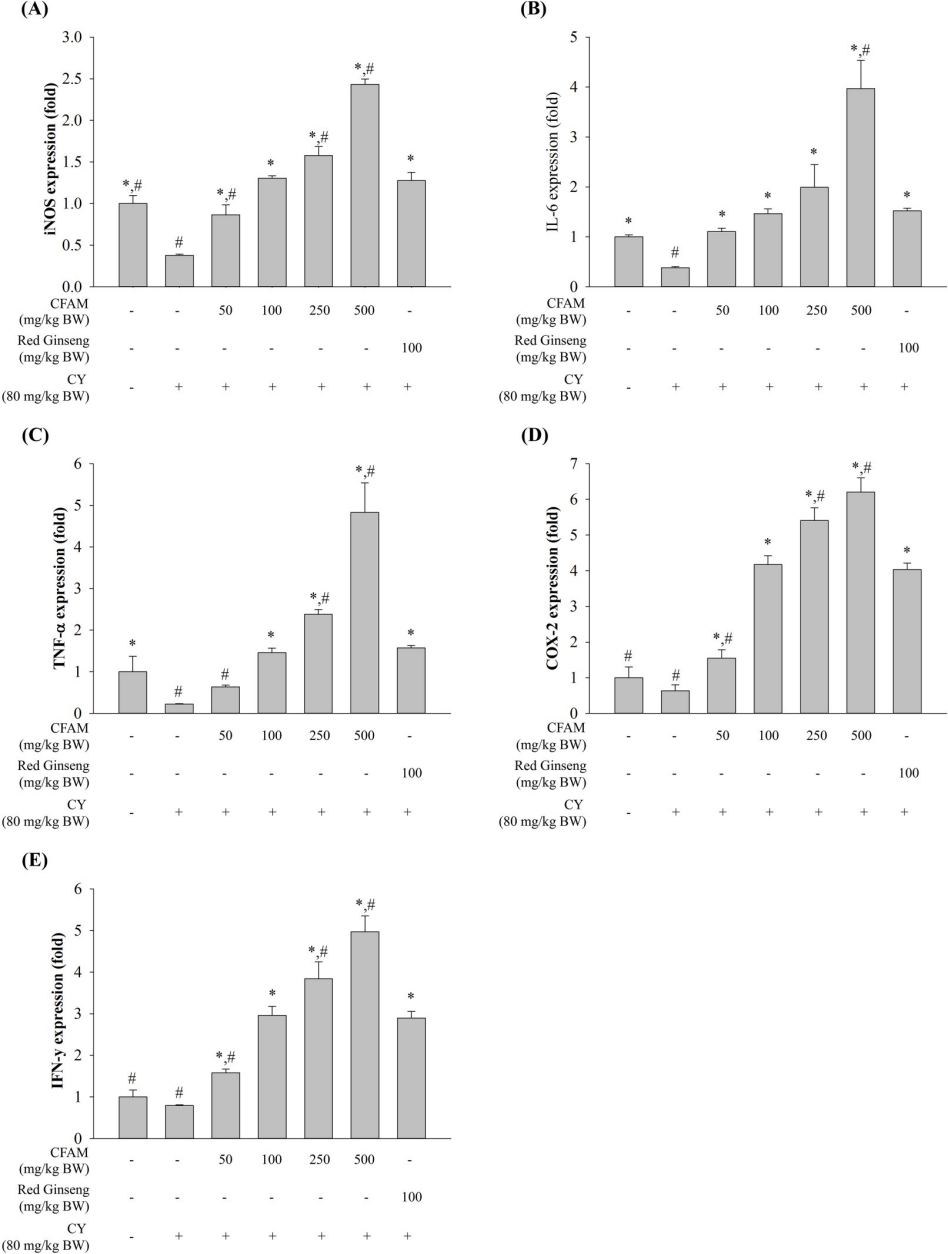
Relative expression (fold-change) of immune genes in LPS-stimulated peritoneal macrophages. Peritoneal macrophages were stimulated with 1 μg/mL of LPS. After 24 h stimulation, total RNA was extracted from the stimulated cells. The cytokine expression was analyzed using the QuantStudio™ 7 FlexReal-Time PCR System. (A) *iNOS*, (B) *IL-6*, (C) *TNF-α*, (D) *COX-2*, and (E) *IFN-γ*. Significant differences were observed at *P < 0*.*05* compared to the saline (*) and ginseng (#) groups.

Immune-associated gene expression in peritoneal macrophages, in addition to that observed in splenic lymphocytes, was analyzed in order to investigate the effect of CFAMs treatment on the activation of peritoneal macrophages from the CY-treated mice. Fig 4 shows that the expression of iNOS, IL-6, TNF-α, COX-2, and IFN-γ was remarkably downregulated in the CY-treated mice. Compared to the CY-treated group, the CFP-treated group showed a dose-dependent and significantly higher expression of these immune-associated genes. Furthermore, peritoneal macrophages treated with 100, 250, and 500 mg/kg BW CFAMs showed similar or higher gene expression than the red ginseng-treated group (positive control).

### Effect of CFAMs treatment on NF-κB and MAPK signaling pathways

**Fig 5:**
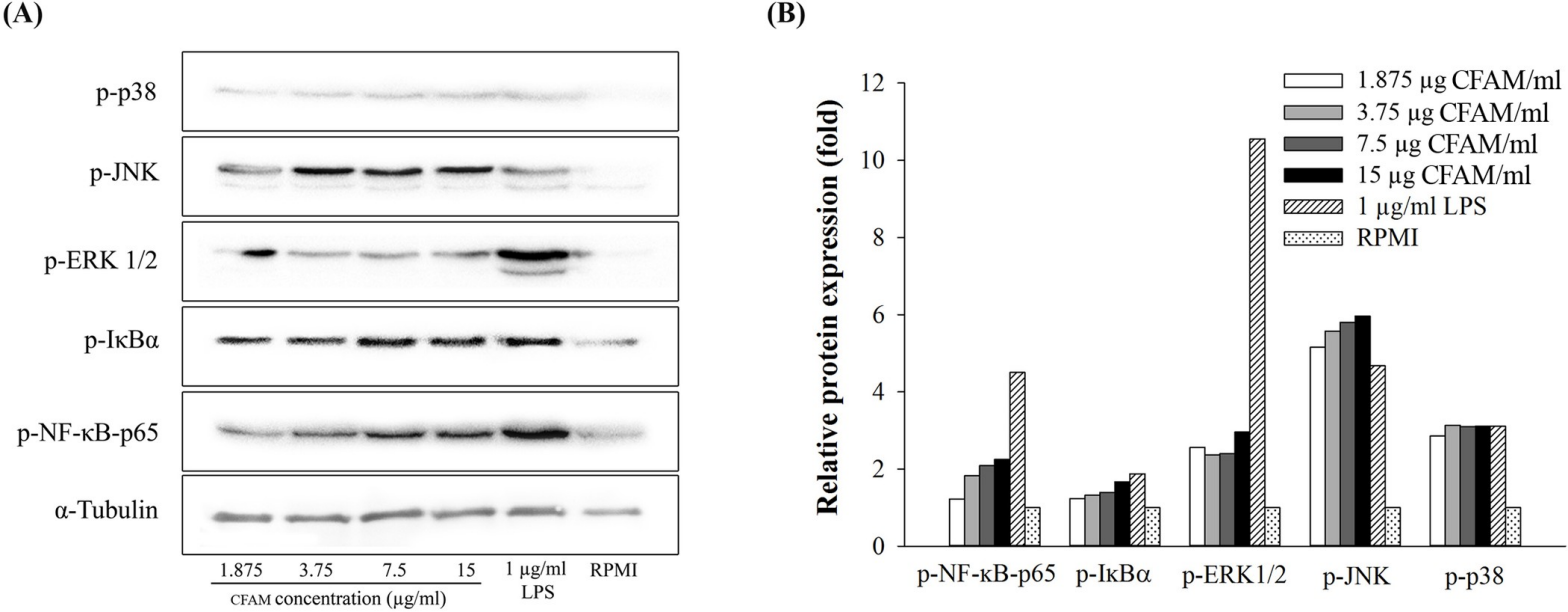
Effects of CFAMs on the proteins associated with NF-κB and MAPK pathways in RAW264.7 cells. Cells were stimulated with various concentrations of CFAMs or 1 μg/mL of LPS for 24 h. Total protein was extracted from each treatment and was separated on SDS-PAGE. The separated protein was transferred on to PVDF membrane. The membrane was incubated with the specific antibodies and was detected using the Pierce® ECL Plus Western Blotting Substrate and were imaged using the ChemiDoc XRS+ imaging system. (A) Western blots showing protein bands, (B) Relative band intensity.

To analyze the mechanism by which CFAMs regulated immune responses in physiological systems, RAW264.7 cells were grown in the presence of varying concentrations of CFAMs. As shown in Fig 5, compared to the negative control (RPMI), CFAMs treatment increased phospholyration of IκBα and NF-κB-p65 in a dose-dependent manner. In addition, phospholyration of ERK, JNK, and p38, which are the key biomarkers for the mitogen-activated protein kinase (MAPK) pathway, was also investigated. It was observed that CFAMs increased the phosphorylation of these proteins in a dose-dependent manner.

### Effect of CFAMs treatment on NF-κB and MAPK activation inhibited-RAW264.7 cells

**Fig 6:**
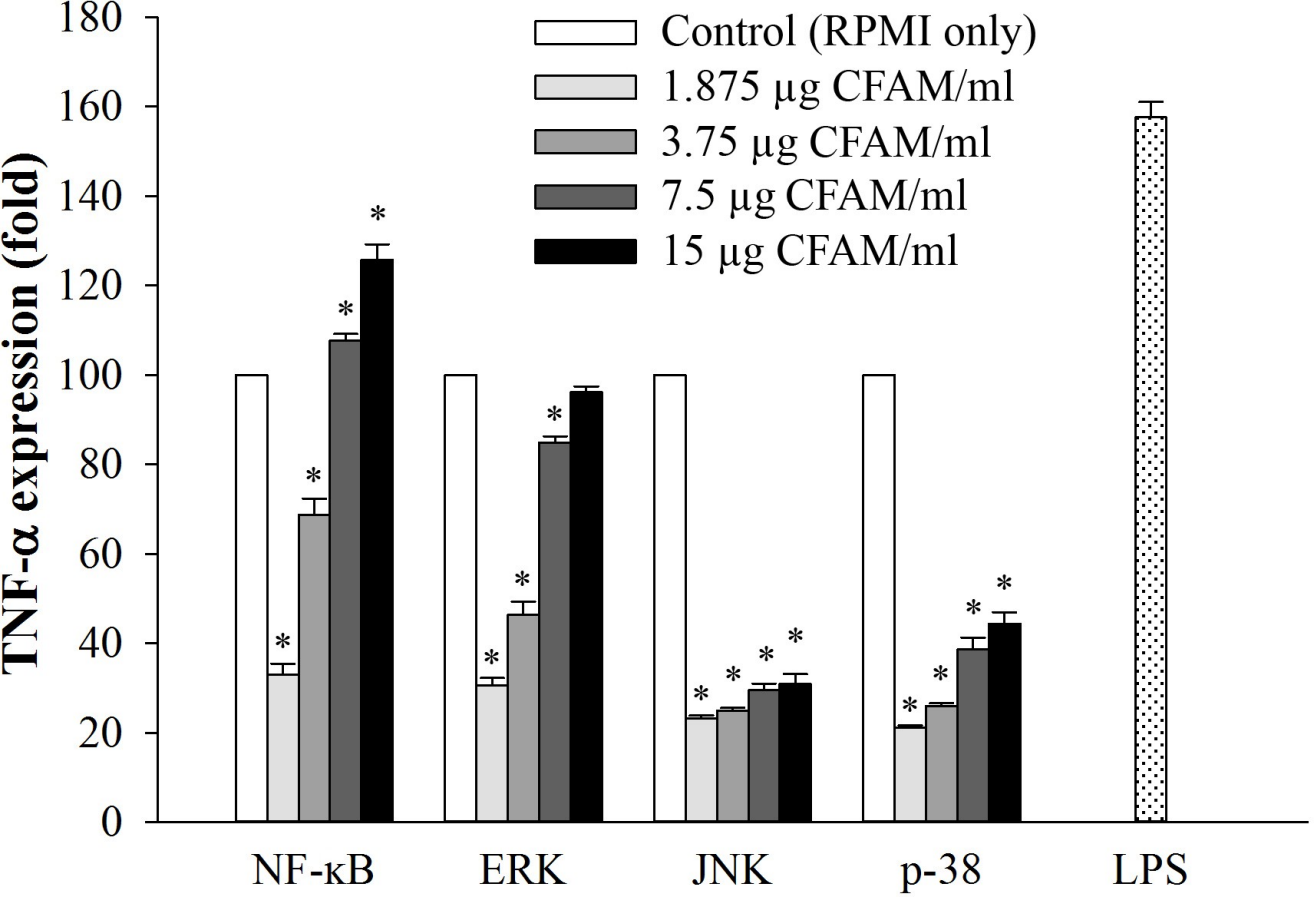
Relative expression (fold-change) of *TNF-α* in CFAM-treated NF-κB and MAPK activation inhibited-RAW264.7 cells. RAW264.7 cells were treated with the f NF-κB activation inhibitor and MAPKs activation inhibitors. After that, these cells were stimulated with various concentrations of CFP or 1 μg/mL of LPS. After total RNA extraction, TNF-α expression was analyzed using the QuantStudio™ 7 FlexReal-Time PCR System. Significant differences were observed at *P < 0*.*01* compared to the control group (*).

In order to investigate the effects of CFAMs on cellular signaling for immune-regulation, TNF-α expression was examined in NF-κB and MAPK activation inhibited-RAW264.7 cells (Fig 6). Compared to the RPMI group (negative control), the CFAMs-treated group showed increased expression of TNF-α in the NF-κB and MAPK activation inhibited-macrophages in a dose-dependent manner. Notably, in the NF-κB and ERK inhibited-RAW264.7 cells treated with high doses of CFAMs (for NF-κB inhibited cells, CFAMs = 7.5 and 15 μg/mL; for ERK inhibited cells, CFAMs = 15 μg/mL), TNF-α expression was restored and similar to or higher than that observed for the RPMI group (negative control).

## Discussions

In humans, immune-regulation is important for protection against several diseases, including tumorigenesis [30]. CY, a well-known chemotherapeutic drug used for tumor treatment, induces adverse effects on the immune system and causes immunosuppression, which can be life threatening [31]. Therefore, many natural compounds having immune-modulation activities have been reported recently. These compounds alleviate the deleterious effects of chemotherapeutic drugs [1, 30–32]. Particularly, polysaccharides with low toxic side-effects have been used as adjuvant medications or immunomodulators for strengthening host defense responses [33, 34]. In the present study, we determined the efficacy of polysaccharides isolated from *C*. *fragile* in enhancing immunological functions in immunosuppressed BALB/c mice.

The spleen is an important lymphoid organ consisting of immune cells, such as monocytes, macrophages, B and T lymphocytes, for mediating immune responses [35]. Spleen-associated factors, such as spleen index and NK cell activities, reflect the immune status of an individual [36]. Especially, lymphocyte proliferation is the most direct indicator of the state of host immunity [37]. T and B lymphocytes are known to react with stimulation activated by antigens or mitogens, which is a common type of non-specific immune regulation [12]. Proliferation of T and B lymphocytes is induced by Con A and LPS, respectively [38]. Fig 1 shows that CFAM accelerated the recovery of spleen size and spleen index in the CY-induced immunosuppressed mice. CFAM also promoted T- and B-cell proliferation and NK cell activities in splenocytes in a dose-dependent manner. Previous studies reported that CY could inhibit both humoral and cellular immune responses [39, 40]. Notably, CFAM-treated mice did not show any mortality. Additionally, compared to the normal group, their body weights also did not change significantly (S1 Fig) [30, 41, 42]. These results indicated that under immunosuppressive conditions, CFAMs treatment establishes immune homeostasis in physiological systems.

Macrophages are immune cells which play important roles in combating infection and removing tumor cells in hosts. They are also closely associated with immune-modulation in cancer therapy [43]. Phagocytes, including macrophages, are important for the regulation of innate immune responses. Phagocytosis is the mechanism by which the phagocytes ingest and kill microorganisms or cancer cells [32]. Increased production of NO by macrophages protects host cells from infection [44]. Macrophages also play an important role as antigen-presenting cells and coordinate with T lymphocytes for regulation of adaptive immune responses [32]. Fig 3 shows that in immunosuppressed mice, CY impaired peritoneal macrophage proliferation and NO production; however, CFAMs enhanced proliferation and NO production of peritoneal macrophages in a dose-dependent manner. Furthermore, CFAMs considerably increased phagocytosis by peritoneal macrophages in a dose-dependent manner, suggesting that CFAMs could enhance non-specific immune functions in CY-treated immunosuppressed mice to improve immune homeostasis.

Several subtypes of T cells, including T helper (Th) cells, cytotoxic T cells, suppressor T cells, effector T cells, are present. Particularly, the type 1 (Th1) and type 2 (Th2) T helper cells play different roles under different immune conditions. Th1 cells are activated during cell-mediated immune responses; however, Th2 cells are stimulated during humoral or allergic responses [45]. Th1 and Th2 cells produce specific cytokines to directly and indirectly control specific immune responses. IL-2, IFN-γ, and TNF-α are regulated by Th1 cells, whereas IL-4, IL-6, and IL-10 are modulated by Th2 cells [46]. Our results showed that cytokine expression in the CFAMs-treated group was higher than that in the CY-treated group. This suggested that CFAMs, unlike in CY-treated group, stimulated the secretion of Th1 and Th2 cytokines to restore immunosuppression and control the dynamic balance between the Th1 and Th2 cells in mice [47].

To regulate immune responses, intermediates of the NF-κB signaling pathway coordinate with inflammatory intermediates, including iNOS, COX-2, and the pro-inflammatory cytokines [48]. In addition, the MAPK signaling pathway involving ERK1/2, JNK, and p38 regulates cellular differentiation and growth by modulating immune responses during stress [49]. MAPK, along with NF-κB, regulates inflammatory cytokine production and related processes [50]. Our results showed that CFAMs increased the phosphorylation of IκBα, NF-κB-p65, ERK, JNK, and p38. Among these, phosphorylation of JNK was the highest. Moreover, it was shown that TNF-α expression, widely used as an experimental model for studying immunomodulatory activities of polysaccharides [36], was affected in the NF-κB and MAPK inhibited cells. In these cells, the expression of TNF-α was observed to be considerably low. These data suggested that CFAMs activated macrophages via the MAPK pathway and involved the phosphorylation of JNK.

## Conclusions

The present study demonstrated that CFAMs, anionic macromolecules from *C*. *fragile*, enhanced the immune responses in CY-treated immunosuppressed mice via peritoneal macrophage proliferation, NO production and phagocytosis by peritoneal macrophages, enhancing macrophage gene expression, increasing splenic lymphocyte proliferation and NK cell activity, spleen index, and splenic lymphocyte gene expression. Additionally, it was shown using macrophages and NF-κB- and MAPK-inhibited cells that CFAMs stimulated macrophages via the MAPK pathway. Consequently, our results might help in elucidating the immune-enhancing mechanisms of CFAMs under immune-suppressive conditions in cancer treatment. In conclusion, CFAMs may be used as a potent biofunctional and pharmaceutical material for enhancing immunity in humans.

## Supporting information

S1 Fig Effects of different concentrations of CFAMs on body weight of mice. (TIF)
