# Supplementary figures and images for: Immune-enhancing effects of anionic macromolecules extracted from Codium fragile on cyclophosphamide-treated mice
Source: PLoS One. 2019 Feb 19;14(2):e0211570. doi: 10.1371/journal.pone.0211570 (PMC6380620; doi:10.1371/journal.pone.0211570)

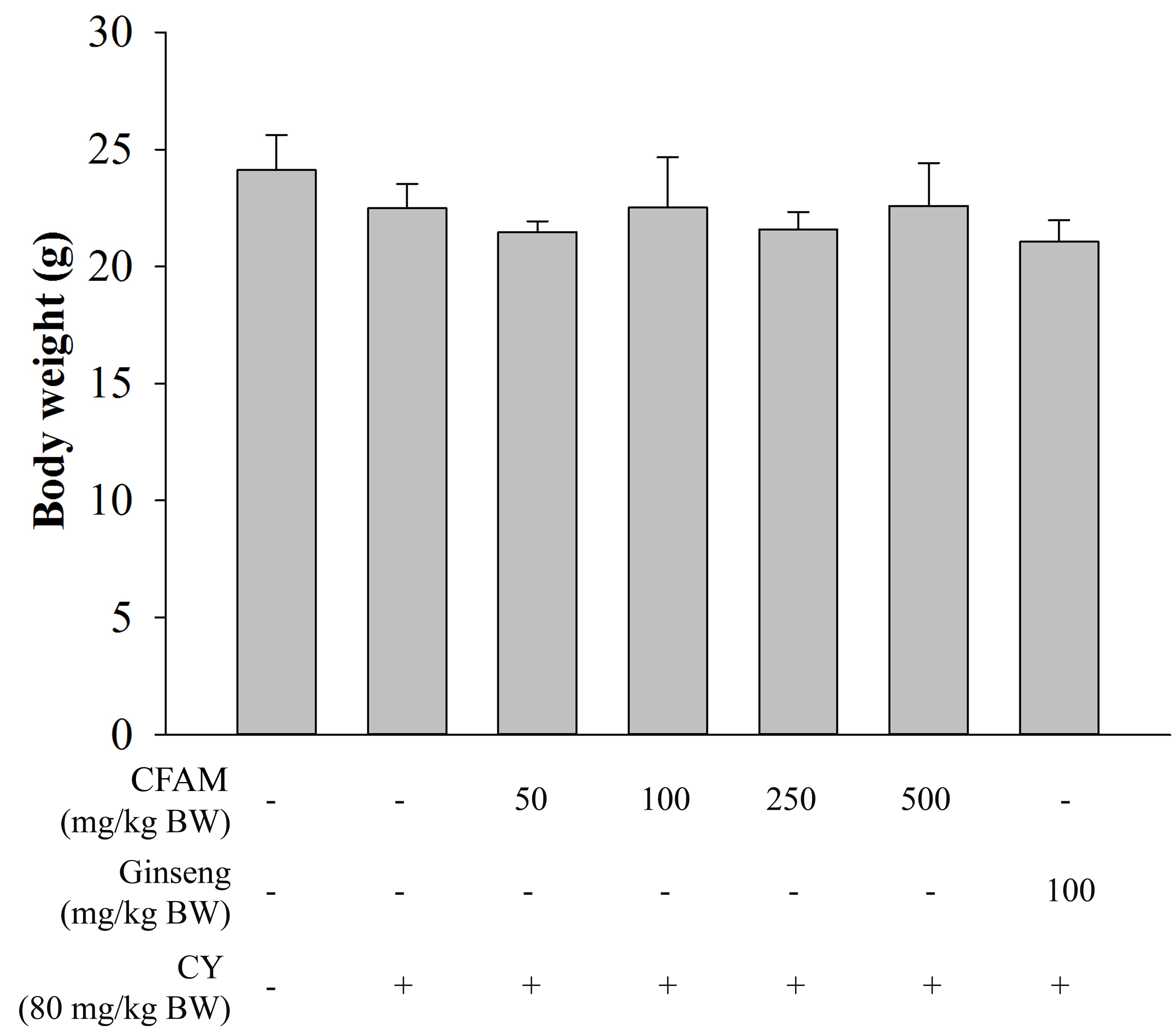

Supplement: S1 Fig — (TIF) [file pone.0211570.s001.tif]
